# Unraveling Muscle Impairment Associated With COVID-19 and the Role of 3D Culture in Its Investigation

**DOI:** 10.3389/fnut.2022.825629

**Published:** 2022-02-10

**Authors:** Maria Luiza G. A. Seixas, Lucas Pari Mitre, Shahin Shams, Gabriel Barbugian Lanzuolo, Cynthia Silva Bartolomeo, Eduardo A. Silva, Carla Maximo Prado, Rodrigo Ureshino, Roberta Sessa Stilhano

**Affiliations:** ^1^Department of Physiological Sciences, Santa Casa de São Paulo School of Medical Sciences, São Paulo, Brazil; ^2^Department of Biomedical Engineering, University of California, Davis, Davis, CA, United States; ^3^Department of Biosciences, Federal University of São Paulo, São Paulo, Brazil; ^4^Department of Biological Sciences, Federal University of São Paulo, São Paulo, Brazil

**Keywords:** skeletal muscle, COVID-19, SARS-CoV-2, inflammation, biomaterials

## Abstract

COVID-19, caused by severe acute respiratory syndrome coronavirus 2 (SARS-CoV-2), has been considered a public health emergency, extensively investigated by researchers. Accordingly, the respiratory tract has been the main research focus, with some other studies outlining the effects on the neurological, cardiovascular, and renal systems. However, concerning SARS-CoV-2 outcomes on skeletal muscle, scientific evidence is still not sufficiently strong to trace, treat and prevent possible muscle impairment due to the COVID-19. Simultaneously, there has been a considerable amount of studies reporting skeletal muscle damage in the context of COVID-19. Among the detrimental musculoskeletal conditions associated with the viral infection, the most commonly described are sarcopenia, cachexia, myalgia, myositis, rhabdomyolysis, atrophy, peripheral neuropathy, and Guillain-Barré Syndrome. Of note, the risk of developing sarcopenia during or after COVID-19 is relatively high, which poses special importance to the condition amid the SARS-CoV-2 infection. The yet uncovered mechanisms by which musculoskeletal injury takes place in COVID-19 and the lack of published methods tailored to study the correlation between COVID-19 and skeletal muscle hinder the ability of healthcare professionals to provide SARS-CoV-2 infected patients with an adequate treatment plan. The present review aims to minimize this burden by both thoroughly exploring the interaction between COVID-19 and the musculoskeletal system and examining the cutting-edge 3D cell culture techniques capable of revolutionizing the study of muscle dynamics.

## Introduction

In December 2019, a novel coronavirus designated as “SARS-CoV-2” emerged in Wuhan, China. A few months after the virus' advent, the World Health Organization (WHO) declared COVID-19, the disease caused by SARS-CoV-2, a pandemic (1). The virus has rapidly spread across the globe, having surpassed 250 million cases and 5 million deaths as of 14 November 2021 (2). SARS-CoV-2 predominantly infects the airways, yielding symptoms related to mild respiratory infections and, in severe cases, acute respiratory syndrome (3). Therefore, the respiratory tract was established as the main SARS-CoV-2 infection focus and, consequently, has been the primary system studied in medical research endeavors.

However, as both the pandemic and scientific research advanced, new SARS-CoV-2 infection hotspots beyond the nasal cavity, throat, and lungs were identified. The blood, brain, heart, and kidneys are examples of systems extensively studied in the context COVID-19 effects. Excessive inflammation, hypoxia, hypercoagulability are among the main reasons that explain these extra-respiratory manifestations of COVID-19 (4–6). Nevertheless, at present, minimal attention has been given to the effect of COVID-19 on skeletal muscle.

As one of the most abundant tissue types in the human body, skeletal muscle is of paramount importance in guaranteeing one's autonomy of movement and independence in daily activities. Damage to the musculoskeletal system is associated with increased Disability-Adjusted Life Years (DALYs) and injury chronification (7), which highlights the importance of skeletal muscle on the quality of life of individuals.

It is now well established that COVID-19 is intrinsically related to several musculoskeletal deteriorating conditions. Clinical observations have revealed that SARS-CoV-2 patients are at high risk of developing **sarcopenia** acutely or insidiously (8), as well as some degree of **cachexia** (9). **Myalgia** has been indicated as the third most common symptom in symptomatic infected patients (10). **Myositis**, **rhabdomyolysis** (9), and **skeletal muscle atrophy** (11) are other disorders deeply associated with the SARS-CoV-2 infection. Lastly, a few neuromuscular disorders have also been reported in COVID-19, including **peripheral neuropathy** (12) and **Guillain-Barre Syndrome** (13). Mechanisms that explain why these disorders commonly develop in the setting of COVID-19 are still lacking, but SARS-CoV-2 induced cytokine storm seems to be one of the main reasons that justify it.

The uncertainty surrounding COVID-19's impact on skeletal muscle has been hampering medical treatment concerning the musculoskeletal system during the pandemic. There is therefore a pronounced necessity to elucidate the mechanisms of SARS-CoV-2 infection and skeletal muscle. In this context, the study of muscle tissue dynamics during both COVID-19 and recovery becomes imperative. The present study, thus, intends not only to investigate deeply how SARS-CoV-2 interacts with and injures the skeletal muscle but also to detail state-of-the-art cell culture methods, mainly 3D cultures, that could be largely used to study muscle dynamics.

## COVID-19 and Its Effect on Skeletal Muscle

### Viral Infections Overview

The study of tissue-specific viral kinetics and replication mechanics is of paramount importance when analyzing the viral impact on each individual tissue. It was recently detected that the differentiation state of skeletal muscle cellular components significantly impacts the ability to resist viral infection in an experimental ZIKV infection model (14). It was suggested that skeletal muscle differentiation could induce changes in cellular factors, predisposing to viral infection in the organ (14). This may be possible due to preferential tropism interactions of specific viruses to replicating cells. Moreover, as fully differentiated cells, skeletal muscle fibers are quiescent, which may lead to partial resistance to viral infection. This phenomenon is well documented in the viral infection setting in regenerative therapy strategies with integrative viruses. Thus, this aspect of modified resistance to certain viral infections may spur an interesting insight when theorizing possible skeletal muscle susceptibility to direct SARS-CoV-2 infection. It is rational to suppose that myoblast (15) would be the primary target of COVID-19, as they are actively replicating, as opposed to myofibers and MuSCs (Muscle Stem Cells), which replicate only under specific conditions. This would further contribute to a loss of regenerative abilities of the skeletal muscle in the vicinity of infection, as myoblasts are essential in the musculoskeletal repair, once they differentiate and donate cellular and nuclear components to structure ruptured fibers (16).

Viruses enter susceptible cells through different mechanisms, such as cell adhesion and interaction with specific receptors, which is followed by invasion through endocytosis and fusion (17). Previous studies of viral diseases have indicated that the mode of cellular infection is related to the tropism of the virus (18–20). Certain viruses, such as HCV, CHIKV, and ZIKV have a tropism for the skeletal muscle or show signs of direct invasion and impairment in anatomopathological studies, such as a few with SARS-CoV-1 and MERS (21, 22). SARS-CoV-1 and MERS infections result in an atrophic profile of skeletal muscle degeneration associated with focal necrosis and Z disc streaming. This report suggests that SARS-CoV-1 and MERS infection impacts skeletal muscle via direct viral invasion. On the other hand, immune cell infiltration, myositis, and macrophages with viral particles confirm a systemic impairment to skeletal muscle secondary to immunological deterioration. The fact that other RNA viruses, such as influenza virus and HCV, may produce skeletal muscle fiber focal necrosis in a similar way sheds light on the importance of studying RNA viruses' mechanism of skeletal muscle impairment (18, 22).

However, tissue-specific harm in the onset of viral infections may not always be distinguishable from systemic inflammation, an indirect impact as they are concurrent phenomena. The age-related immunological decline, for instance, marked, among other characteristics, by loss of phagocytic function and loss of MuSCs and FAP (fibroadipogenic progenitors) proliferation, is a central factor that hinders distinction between both phenomena (18). For example, it has been demonstrated that clinical musculoskeletal deficiencies and myofiber stress are still present in HIV patients, even after viral levels are undetectable (23). Thus, viral infections, such as HIV, exemplify how skeletal muscle can be impaired either through direct or systemic conditions.

### SARS-CoV-2 Investigation: SARS-CoV-2 Mechanism of Action and Skeletal Muscle

#### Viral Entrance

SARS-CoV-2 is a positive-sense single-stranded RNA virus, member of the coronavirus family. Cellular entry mechanisms of coronaviruses hinge on the binding of spike (S) protein to a specific cellular receptor, which triggers S protein priming by cellular proteases (24), a process that allows further viral spread.

On mature viruses, the S protein is a trimer composed of three receptor-binding S1 heads placed on top of a trimeric membrane fusion S2 stalk. S1 carries a receptor-binding domain (RBD) that recognizes angiotensin-converting enzyme 2 (ACE2) as its receptor (25). Interestingly, RBD's conformation constantly changes in order to favor virus activity in host cells: it acquires a standing-up position to allow receptor binding and a lying-down position to allow immune evasion (26). Further membrane fusion between the host cell and the virus occurs by a specific cleavage mechanism, mediated mainly by transmembrane serine protease 2 (TMPRSS2) and lysosomal proteases cathepsins (25): S protein is activated proteolytically at the S1/S2 boundary, which prompts S1 dissociation and S2 structural change (27, 28). Then, viral replication inside cells begins to occur (11).

The ACE2 receptor is widely expressed in the human body and the musculoskeletal system is not an exception. It has even been reported that the heart and skeletal muscle express ACE2 abundantly (29), which would raise the possibility of direct musculoskeletal damage seen in COVID-19 (which will be further discussed in detail) being intrinsically associated with ACE2 expression in the tissue.

The receptor is a multifunctional type I transmembrane metallocarboxypeptidase. Besides acting as a receptor for SARS-CoV-2 entrance into human cells, it is also constitutionally associated with the renin-angiotensin-aldosterone system (RAS), responsible for blood pressure regulation (11). ACE2, due to its role in RAS and in other pathways that will be further outlined, acts as a protective component in the tissues where it is expressed. The binding of SARS-CoV-2 to ACE2 would thus impair the protective role of the receptor, which could, once more, contribute to the musculoskeletal damage seen in patients that experience COVID-19.

The need to study the SARS-CoV-2 mechanism of action in humans to combat the spread of the pandemic spurred the scientific community to compare the viral kinetics to other coronaviruses' kinetics, mainly SARS-CoV-1, with the principal aim of identifying possible uncovered processes that could support COVID-19 studies and clinical outcomes. In this context, the first key difference that was identified between both coronaviruses is associated with RBD. It is known that RBD conformation is dynamic, that is, it alters between two polar states of interaction with ACE2 (25). Interestingly, when comparing both viruses, SARS-CoV-1 was indicated as having a higher frequency of RBD in the standing-up position than SARS-CoV-2. This conformation allows the S1 spike subunit to be available for interaction and adhesion to human ACE2, which is, in turn, associated with infectivity. On the other hand, the affinity of RBD-human ACE2 binding was suggested to be higher in SARS-CoV-2. These apparently paradoxical results translate mechanistically to an enhanced immune evasion of SARS-CoV-2: the main reasons being that in SARS-CoV-2, on top of the fact that RBD is less exposed when compared to SARS-CoV-1's RBD, it spends more time in the lying-down conformation, which makes it less detectable by immune cells and antibodies. This phenomenon alone would result in reduced infectivity of SARS-CoV-2. However, it is countered with a higher affinity of RBD-human ACE2 binding, which would enhance virus entry and compensate for immune evasion (25). As a consequence, on the whole, it is possible to conclude that SARS-CoV-2 has higher infectivity, as it is more efficient in perpetuating infection once in the human organism.

This rationale is similar for other coronaviruses, such as MERS-CoV, because S protein has been reported as its major surface antigen, and its RBD is endowed with the same flexibility, experiencing the same conformational changes described previously for SARS-CoV-1 and SARS-CoV-2 (26, 30). The presence of flexible RBD in coronaviruses is worrisome by itself, as it practically guarantees virus entry once receptor interaction is achieved (26). In conclusion, the particularities of SARS-CoV-2, a member of an already successful family of infective coronaviruses raise red flags as to a more efficient, and thus potentially harmful, mechanism of viral infection.

### ACE2 and RAS

The ectodomain of ACE2 is primarily responsible for cleaving angiotensin II (Ang II) into Angiotensin 1-7 (Ang 1-7). As a multiple intracellular signaling pathway mediator, Ang 1-7 stimulates vasodilation, anti-proliferation, anti-inflammation, and anti-fibrosis processes: production of nitric oxide primarily via the AKT-eNOS pathway, inhibition of MAP kinase signaling (p38, ERK1/2, and JNK), suppression of reactive oxygen species (ROS) production by NADPH oxidases, inhibition of transforming growth factor beta (TGF-β)-SMAD signaling, and modulation of cAMP signaling response. The fact that Ang 1-7 binds to Mas, MrgD (a member of Mas-related G-protein coupled receptors), and Ang II type 2 receptors allows it to exert its effect. It has also been suggested that alamandine, an endogenous peptide that is cleaved from angiotensin A by ACE2, binds to MrGD and provides protection similar to Ang 1-7 (31).

Under different pathological conditions, the ectodomain of ACE2 is shed by a disintegrin and metalloproteinase 17 (ADAM17), also known as tumor necrosis factor-alpha (TNFα)-converting enzyme (TACE). This shedding process impairs ACE2 activity. In certain situations, such as inflammation, ADAM17 is increased (32), which would lead to decreased ACE2 activity in the setting of inflammatory states. The diminished activity of ACE2 would impair the protective role of the receptor, but, at the same time, could protect against COVID-19 infection, due to the fact that the virus enters the cell by ACE2 receptors. Further studies are required to investigate this dynamic.

In some comorbidities, such as diabetes, a systemic lower expression of ACE2 has been described (33). At the beginning of the SARS-CoV-2 pandemic, it was thought that this could represent protection once it meant fewer entrance receptors for the virus (33). Nevertheless, while the virus encounters fewer ACE2 receptors, the organism is also characterized by a suppressed ACE2-Ang1-7-Mas axis and an exacerbation of ACE-Ang II-AT1 axis, which ultimately represents a pro-inflammatory, pro-fibrosis, and pro-proliferation activity over an anti-inflammatory, anti-fibrosis, and anti-proliferation activity (34). Therefore, diabetic patients not only experience a state of low ACE2 because of their condition but also present a characteristic ACE2 downregulation due to virus entrance, which could, among other factors, contribute to why those patients have a worse COVID-19 prognosis than non-diabetic patients.

### ACE2, Ang II, and Ang 1-7 in Skeletal Muscle

Ang II has been reported to hinder muscle protein synthesis mainly by inhibiting the insulin growth factor 1-AKT-mammalian target of Rapamycin (IGF-1-AKT-mTOR) pathway, responsible for autophagy inhibition, energy homeostasis and structural plasticity (35). Ang II has also been shown to induce the up-regulation of atrogenes, especially MuRF-1 and atrogin-1, which are mainly responsible for ubiquitin-proteasome-dependent protein breakdown and caspase-dependent myonuclear apoptosis. These processes spur the production of NOX2-dependent ROS production and induce NfκB-dependent inflammation and mitochondrial damage, which contribute to muscle fibrosis (34).

Ang 1-7 exerts a protective effect on skeletal muscle mainly due to the inhibition of Ang-II mechanisms of atrophy and fibrosis induction (34). Experimental Ang 1-7 infusions relieved muscle dysfunction in a few conditions, such as cancer cachexia (36), elevated concentrations of Ang II (37), atrophy induced by disuse (38, 39), and muscular dystrophy (40, 41). Conversely, it was seen that ACE2 overexpression and ensued augmented Ang 1-7 production that favors muscle dystrophy (42). However, the mechanism is yet to be confirmed (34).

Considering that SARS-CoV-2 entrance in cells occurs through ACE2 receptor, COVID-19 was already associated with a downregulation of ACE2 and a subsequent **increase in Ang II** levels and **decrease in Ang 1-7** (43). Thus, on the whole, skeletal muscle in SARS-CoV-2 infected patients might be more susceptible to muscle damage.

#### SARS-CoV-2 Induced Inflammation

Upon entrance into the organism and its cells, it is known that SARS-CoV-2 triggers an aggressive inflammatory response that results in damage to multiple structures (44). Contrastingly, inflammation can act as a stimulating agent for skeletal muscle tissue recovery after lesion through a series of mechanisms (45, 46). This dynamic depends on the interaction between cellular, matrix, and circulatory components that orchestrates this process, maximizing the potential of recovery. During the SARS-CoV-2 course of infection, what is seen in terms of systemic pro-recovery inflammation state is disrupted. It has already been established that the entry of the virus through the respiratory airways prompts a strong response to infection, provoking phenomenons of dysregulated immunological response, such as the cytokine storm (11). The cytokine storm is a potentially fatal condition caused by excessive liberation and activation of chemoattractant and inflammatory cytokines, products of interaction between the virus and the host's immune system (47). The storm of cytokines is also characterized by lymphopenia and excessive mononuclear cell infiltration in multiple tissues (47, 48). The enormous amount of inflammatory markers in the COVID-19 patient's blood have been linked to increased disease severity and hyperinflammation and increased neutrophil/lymphocyte ratio, with possible depletion of circulating T cells.

**Figure 1:**
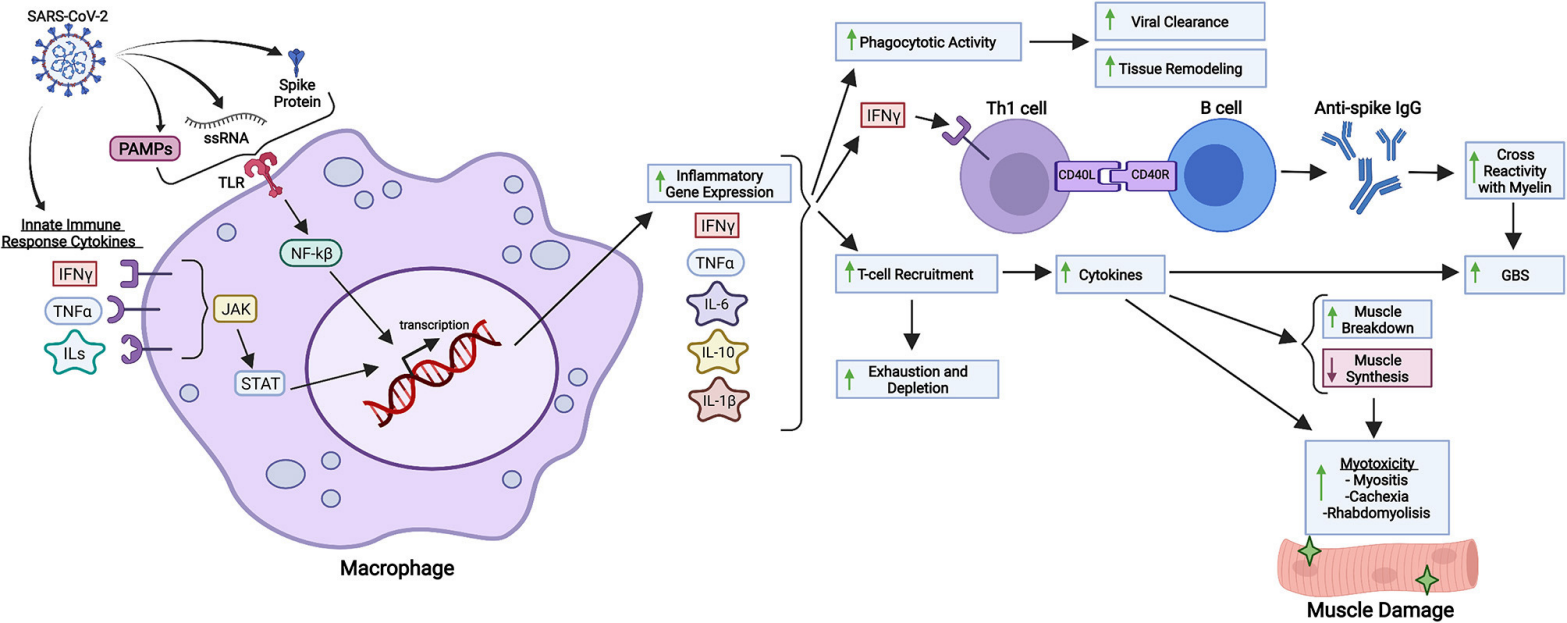
Inflammatory and immunological repercussions of SARS-CoV-2 infection. SARS-CoV-2 components such as the S protein, PAMPs and viral single-stranded RNA (ssRNA) are recognized by macrophages and other immunological cells through Toll-Like Receptors (TLR), triggering intracellular cascades responsible for initiating inflammatory gene expression and cytokine production. Cytokines from the innate immune response against COVID-19 also intensify inflammatory gene expression through different pathways, such as JAK-STAT signaling. Increased synthesis of IL-6, IL-10, IL-1ß, IFN-γ and other cytokines in response to viral infection enhance phagocytotic activity, B cell class differentiation into IgG synthesizing plasmocytes and tissue-specific T-cell recruitment. Phagocytotic activity is related to viral clearance and tissue remodeling as a response to chronic inflammation. Anti-spike IgG might cross-react with neuronal myelin, predisposing SARS-CoV-2 infected patients to GBS (Guillain-Barré Syndrome), a disease whose development is also associated with cytokines produced in the setting of SARS-CoV-2 infection. Lastly, T-cell recruitment is associated with increased production of cytokines, which not only enhance myotoxicity through decreased muscle protein synthesis and increased protein degradation, but also deplete circulating T cells, which can aggravate systemic immunological response to infection. Created with Biorender.com.

Possible mechanisms associated with T-cell depletion may be due to intense tissue recruitment as a consequence of excessive cytokine signaling (Figure 1). It is important to stress that immunological virus combating involves both innate and adaptive immune responses, and it is logical to expect disturbances in both domains when infections occur. It is possible for different organisms to have different types and intensities of innate/adaptive immune response based on each individual profile of response, and this may alter due to age and the development of medical conditions (49). People that tend to present mostly with the adaptive cellular response profile might have a stronger Th2 over Th1 response, which is especially true in infants. This has been correlated with a higher frequency of respiratory viral infections in children (not necessarily with disease severity) due to decreased production of cytokines crucial for viral clearance, such as IFN-γ (49, 50). In addition, viral clearance activity can occur without clinically detectable IgM and IgG, as phagocytic immune cells rapidly clear infected cells - impacting multiplicity of infection. It is possible to conclude that there are numerous possibilities when it comes to immunity development regarding SARS-CoV-2 infection, which has been proven by the myriad of clinical cases detected and mechanisms elucidated.

Possible hyperactive pathways related to pathological inflammation such as IL-6 signaling, TLR4–TRIF signaling, JAK–STAT signaling, CCR5, Complement component C5, and others are currently being investigated, but they have been solidly correlated hyperinflammation due to SARS-CoV-2 infection. These pathways, which mediate the response through the cytokines IFN-α/γ, IL-1ß, IL-6, IL-12, TNF-α, C-reactive protein (CRP), and components such as ferritin constitute proinflammatory routes that promote macrophage and natural killer (NK) cell activation, T cell recruitment, transcription of Interferon Stimulated Genes (ISGs) and inflammatory genes, inflammatory vascular mechanisms and immunological feedback loops; all phenomenons linked and potentially pathologically hyperactive in SARS-CoV-2 infection (47, 51–53) (Figure 1). It is interesting to observe that these major inflammatory pathways all derive from immunological mechanisms related to virus sensing through viral single-stranded RNA recognition, immunological recruitment, and activation subsequential to proinflammatory stimuli such as pathogen-associated molecular patterns (PAMPs), and overall increased cytokine production and liberation (47, 54). In conclusion, all prerequisites for harmful inflammatory cascade activation are fulfilled by SARS-CoV-2 (Figure 1) and other viruses such as SARS-CoV-1 and MERS. In general, studying the immunological response to viruses provides a powerful perspective as to how viruses can trigger systemic inflammation.

### Main Musculoskeletal Complications Due to COVID-19

#### Sarcopenia

It has become clear to the scientific community that patients that survive COVID-19 are at increased risk of acute sarcopenia and underlying muscle insufficiency (8). This is due to diminished ACE2 presence, inflammation, prolonged bedrest along with diminished physical activity, and hypoxia.

#### ACE2 and Skeletal Muscle

Several studies suggest that ACE2 plays a protective role against sarcopenia. Mice deficient in ACE2, G protein-coupled Apelin receptor (APLNR) and apelin have been shown to manifest accelerated sarcopenic phenotypes. These ACE2-knockout mice also exhibited a significant decrease in the essential amino acid tryptophan (Trp) in plasma and organs, including skeletal muscle (34). Considering that serum concentration of Trp was already associated with an increase in the volume of skeletal muscle in patients that present with diffuse large B-cell lymphoma (55), it is possible to state that ACE2-related-Trp-reduction may contribute to skeletal muscle volume diminishment, a clinical feature in sarcopenia. The same study also detected smaller tibialis anterior fiber diameters in Trp-deficient mice, comparing them to control mice (55).

**Figure 2:**
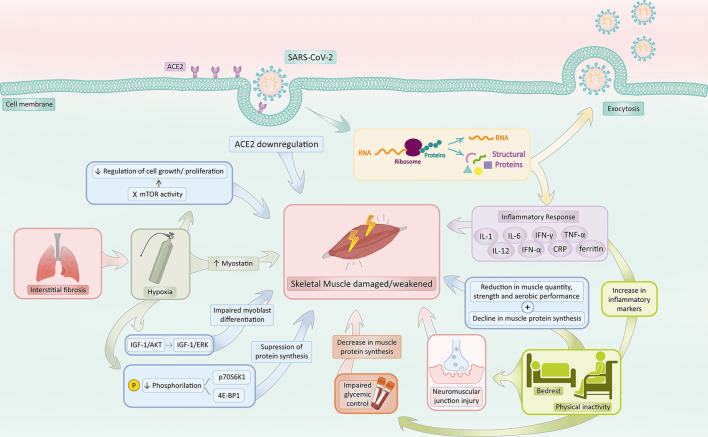
COVID-19 associated sarcopenia mechanisms. SARS-CoV-2 infection activates a series of mechanisms responsible for increasing the risk of sarcopenia development. COVID-19 induces **ACE2 downregulation** and hinders the ACE2 protective effect, predisposing skeletal muscle to sarcopenia. The **inflammatory response** in response to infection, mainly characterized by the production of IL-1, IL-6, IFN-γ, TNF-α, IL-12, IFN-α, CRP, and Ferritin, prompts muscle catabolism, reducing muscle protein synthesis and increasing its susceptibility to damage and death. **Bedrest and physical inactivity** in the context of patients with COVID-19 not only increases several inflammatory biomarkers (such as TNF-α, CRP, and IL-6) but also reduces muscle quantity, strength, anaerobic performance, and protein synthesis. Additionally, the inactivity state impairs glycemic control, which also contributes to a decrease in muscle protein synthesis and damages neuromuscular junctions. **Interstitial pneumonia** secondary to COVID-19 commonly triggers a state of **hypoxia**, which accounts for (1) a decrease in phosphorylation of p70S6K1 and 4E-BP1, (2) a switch from the IGF-1/Akt to IGF-1/ERK signaling pathway, (3) an increase in myostatin levels and (4) an inhibition of mTOR. The decrease in phosphorylation of p70S6K1 and 4E-BP1 suppresses protein synthesis. The switch from the IGF-1/Akt to IGF-1/ERK signaling pathway impairs myoblast differentiation. Elevated levels of myostatin activate catabolism and negatively regulate muscle growth. Inhibition of mTOR activity hampers the regulation of cell growth and proliferation, which augments the risk of damage within the skeletal muscle. Hypoxia, thus, makes the musculoskeletal system susceptible to sarcopenia. Created with adobe.com/illustrator.

COVID-19 has been associated with a downregulation of ACE2, partially motivated by viral infiltration through ACE2 receptors (43). Consequently, considering the sarcopenia-related effects of ACE2 absence, the reduction in ACE2 in COVID-19 patients would contribute to sarcopenia (Figure 2).

#### Inflammation

It has been suggested that sarcopenia is intrinsically related to impaired immunity, as it was reported that the condition is associated with diminished proliferation of peripheral mononuclear cells, augmented ratio of neutrophils to lymphocytes, and damaged homeostasis of NK cells, which contributes to a state of immune senescence. The principal reason that justifies the **impaired immunity** in patients that experience sarcopenia is the presence of abnormal myokines, mainly IL-15, IL-17, and IL-6, responsible for modulating the proliferation and function of innate and adaptive immune cells (56). Along with immunity alterations, patients with sarcopenia also present with a state of **metabolic stress**, characterized by skeletal muscle catabolism with the aim of providing the immune system, liver, and gut with amino acids, mainly glutamine (56).

SARS-CoV-2 infection is initially characterized by a local inflammatory response that triggers the production of cytokines such as interferon-α, interferon-γ, IL-1, IL-6, IL-12, TNF-α, CRP, and monocyte chemotactic protein-1 (56, 57). Inflammation in the setting of COVID-19 was also reported to be associated with elevated metabolic stress and muscle catabolism (56) (Figure 2), along with other symptoms, such as fever, cough, fatigue, headache, hemoptysis, and diarrhea (57).

As COVID-19 associated inflammation progresses, a process of systemic inflammation sets in, causing more severe damage to the organism (58). Concerning the inflammatory response in the disease, special attention has been given to the production of proinflammatory cytokines, mainly TNF-α, IL-1, IL-6, and CRP, one of the main acute phase reactants. Ferritin, another acute phase reactant, has also been reported to be elevated in infected patients (57).

**TNF-α**, for instance, negatively affects muscle protein synthesis (Figure 2), as it modifies Eukaryotic translation initiation factor 4E (eIF-4E) availability, which results in a reduction translational efficiency of Messenger Ribonucleic Acid (mRNA). The consequence of this process is the emergence of an anabolic resistance state, which demands a higher protein absorption to prompt muscle protein synthesis (59). Skeletal Muscle resistance to anabolic stimuli is a marker of muscle impairment in chronic degenerative and traumatic skeletal muscle diseases (60, 61).

**Ferritin**, an acute phase reactant also elevated in COVID-19, has also been indicated as a compound that interacts with the production of energy in mitochondria, enhancing anaerobic metabolism as opposed to aerobic metabolism, which intensifies ROS generation and increases the susceptibility of cells to damage and death (Figure 2) (8).

The generally high level of inflammation experienced by patients infected by SARS-CoV-2 also makes the human body intensely vulnerable to multi-organ damage, a process hallmarked by endothelial harm and a predisposition to thrombosis, which greatly affects the musculoskeletal system (Figure 2) (8).

In summary, it is highly likely that inflammation triggered by SARS-CoV-2 infection acutely impacts skeletal muscle physiology and function (Figure 2) (8).

#### Bedrest and Physical Activity

The extensive morbidity associated with COVID-19 may also predispose those affected to sarcopenia. Patients that experience the disease undergo prolonged periods of bed-rest as well as diminished physical activity and prevention of mobilization outside of ward areas or side rooms inside some hospitals (59). It is also important to note that immobilization due to COVID-19 hospitalization has been reported as more severe in comparison to other conditions such as limb fracture and severe pneumonia. As a consequence, these individuals face not only a reduction in muscle quantity, strength, and aerobic performance but also declines in muscle protein synthesis due to a modified expression of ubiquitin ligases (MuRF-1 and MAFbx) (Figure 2) (59).

It has been determined that patients hospitalized with COVID-19 experience rapid and dramatic muscle loss. Within the first 7 days of hospitalization, patients can experience a median decrease of 18.5% in the area of the rectus femoris muscle (8). Furthermore, chronic inactivity stimulates denervation of muscle fibers and neuromuscular junction (NMJ) injury, which has been demonstrated by an increment in NCAM (neural cell adhesion molecule)-positive muscle fibers in three separate bedrest studies lasting 3, 10, and 15 days. As a glycoprotein usually expressed only in embryogenesis, hence absent in adult muscle, an increase of this protein in adult muscle indicates a denervation or reinnervation process in progress (Figure 2) (62).

Length of hospital stay and the severity of a patient's COVID-19 disease state are, therefore, intrinsically linked, as demonstrated by a translation of the GLISTEN study (63) to COVID-19, which reported that 11 days of COVID-19 hospitalization was associated with a 38.4% unadjusted increase in the risk of sarcopenia (8).

COVID-19-related social distancing measures and lock-down and have also contributed to the extensive inactivity that individuals have been recently facing. The Effects of home Confinement on multiple Lifestyle Behaviors during the COVID-19 outbreak (ECLB-COVID19), a large electronic survey that was applied around the world and investigated the effects of lock-down measures during the first wave of COVID-19 in 1,047 people, detected a decrease in physical activity by and an increase in sitting time from 5 to 8 h per day (8). The inactivity style may predispose individuals to a decrease in insulin sensitivity and an increase in visceral fat, as reported in a study on healthy young men who performed little physical activity (29). These individuals have their normal metabolic arrangement unstabilized and, as a consequence, a reduction in insulin-provoked muscle Akt phosphorylation and in muscle protein synthesis ensues (Figure 2). Additionally, studies have shown that diminishing physical activity is associated with an elevation in inflammatory markers, mainly TNF-α, CRP, and IL-6 (Figure 2) (29). In summary, it has been demonstrated that acute physical inactivity holds an intrinsic correlation with (1) impaired glycemic control, which reduces insulin-provoked muscle Akt phosphorylation, (3) inflammation augmentation, which predisposes the musculoskeletal system to intense damage (64) and (4) reduced muscle protein synthesis, as shown in Figure 2 (29).

#### Pulmonary Involvement of COVID-19 on the Development of Sarcopenia

It has been reported that SARS-CoV-2 begins its infection in the respiratory tissue. As a high-ACE2 and TMPRSS2-expression tissue, viral invasion progresses swiftly, which commonly causes viral interstitial pneumonia correlated with hypoxia (8). Hypoxia, in turn, triggers a string of adaptive responses, the main of which is a down-regulation of processes that demand elevated energy consumption, such as protein synthesis. A suppression in protein synthesis by multiple mechanisms, therefore, occurs. Firstly, there is an inhibition of the mTOR activity: Regulated in development and DNA damage responses-1 (REDD1), a hypoxia-inducible factor-1 target gene, and the sensor of cellular energy balance AMPK acts upon the mammalian target of rapamycin complex 1 (mTORC1) signaling during hypoxic stress, inhibiting it through phosphorylation of tuberous suppressor complex 2 (TSC2) (65). mTOR is part of the phosphatidylinositol 3-kinase (PI3K) cell survival pathway, which controls nutrient availability, mitotic signaling as well as cellular energy and oxygen concentration; therefore, mTOR is essential for the regulation of cell growth and proliferation (66). Decreases in the phosphorylation of Ribosomal protein S6 kinase beta-1 (p70S6K1) and eIF4E-binding protein 1 (4E-BP1) also occur, which reduces mRNA translation activity and inhibits protein synthesis - thus, impairing muscle development (65).

Hypoxia is also associated with higher levels of myostatin (8), an endogenous component of catabolic pathways in muscle cells and a negative regulator of muscle growth (67). Thus, greater concentrations of myostatin are associated with muscle degradation. Furthermore, hypoxia has been related to a switch from the IGF-1/Akt to IGF-1/ERK signaling pathway, which stimulates myoblast proliferation over differentiation, though myogenesis is still present (8). Observational studies with individuals exposed to high altitude hypoxia in mountaineering expeditions have also detected a reduction in muscle mass (68) and muscle fiber size, independently of physical activity levels (69), which confirms the relationship between hypoxia and skeletal muscle impairment.

In conclusion, it is possible to state that COVID-19 predisposes individuals to sarcopenia in multiple ways. The cytokine storm, physical immobilization, and hypoxia commonly present in SARS-CoV-2 infection are the key mechanisms that justify this predisposition. However, further studies are still required not only to confirm this hypothesis but also to investigate uncovered processes possibly contributing to sarcopenia in COVID-19.

#### Cachexia

Cachexia is a complex metabolic syndrome characterized by loss of muscle and associated with underlying illness (70). It is diagnosed upon clinically significant weight loss (71). In COVID-19, cachexia is diagnosed when there is ≥5% weight loss, functional status is impaired, and metabolic derangement (e.g., inflammation) can be documented. Other important factors that allow identification of the disease are: anorexia, low albumin, inflammation and increased muscle protein breakdown (72). There have been a few reports of cachexia secondary to COVID-19, particularly in cases of prolonged illness, which highlights the importance of studying the condition and its interrelation with SARS-CoV-2 infection (71).

Weight loss in COVID-19 is mediated by different mechanisms. First, the expression of acute-phase inflammatory proteins such as fibroblast growth factor, TNFα, ferritin, CRP, IL-factors, interferon-γ, Nuclear factor kappa-light-chain-enhancer of activated B cells (NF-kB), disturb tissue homeostasis, leading to dysregulation of metabolism and proteolysis (73). Second, there is the malnutrition commonly seen in patients with COVID-19. Appetite loss, ageusia, fever, and sedation in SARS-CoV-2 infection are the main factors contributing to malnutrition development (71). Along with that, the potential of SARS-CoV-2 to attack the mucosal epithelium in the gastrointestinal system is also an important element that explains malnutrition in COVID-19 (74). Thirdly, immobilization amid COVID-19 might also be associated with muscle wasting and weight loss (71). The elevated administration of sedatives and opioids aimed at facilitating mechanical ventilation in patients infected by SARS-CoV-2 can also play a role in weight loss and cachexia predisposition. Taking this all into consideration, a catabolic overdrive state ensues, triggering progressive weight loss in COVID-19 (71). Therefore, it is possible that patients that experience COVID-19 are at high risk of developing cachexia, which highly predisposes individuals to mortality and disability. There are still not enough studies that determine the exact relationship between cachexia and COVID-19. This dearth hampers differentiation between both clinical conditions and thus, adequate treatment of them both. In view of this, it is of extreme importance that deeper studies on the relation between cachexia and COVID-19 be developed.

#### Myalgia

Myalgia is one of the most common symptoms of COVID-19, along with fever, cough, and sore throat. Prevalence of the condition amid SARS-CoV-2 infection, identified in a meta-analysis of clinical characteristics was 35.8% (75). Another study, aimed at investigating the olfactory and gustatory function in patients experiencing COVID-19 found that more than 50% of them had myalgia (76). Myalgia is a very common and non-specific symptom, generally defined as muscular pain (77). Muscle-related pain, in turn, is usually associated with a local hypersensitivity (hyperalgesia) to mechanical stimuli (78).

Myalgia usually presents due to generalized inflammation and cytokine response (79). The condition is commonly mediated by IL-6, whose upregulation triggers muscle and joint pain through activation of peripheral nociceptors (80). It was demonstrated in animal models that IL-6 induced hyperalgesia is probably mediated by an increase in pro-inflammatory cytokine production (mainly IL-1ß and TNFα), resident cell activation, polymorphonuclear cell infiltration, prostanoids and sympathomimetic amines release and activation of different intracellular signaling pathways, namely PLA2, PLC–PKC, PKA, PI3K, and MAPKs (78). Of note, IL-6 has been shown not only to elicit acute muscle pain but also to evoke chronic muscle pain, which indicates that it plays an important role both in acute and chronic muscle pain (78).

It has been suggested that myalgia in SARS-CoV-2 infected patients reflects the generalized inflammatory state and the cytokine storm generated in the disease (80). Considering that COVID-19's cytokine storm presents with an elevation of IL-6, TNF-a, and IL-1ß and that pathophysiology of myalgia also involves these cytokines, it seems reasonable to associate SARS-CoV-2 infection with myalgia. It has also been proposed that muscle involvement in COVID-19 may stem from deoxygenation of the musculoskeletal system: surplus cell damage in SARS-CoV-1-infection may increase lactate levels in the human body. Hyperlactemia, in turn, impairs (1) aerobic respiration and ATP synthesis and (3) oxygen-carrying capacity of erythrocytes - predisposing tissues to hypoxia. Reduced ATP production leads to pain and fatigue. Decreased oxygen transportation may induce an ischemic state in muscles during COVID-19 infection (81). Hypoxic ischemia elicits a state of increase in growth factors, cytokine levels, and microvascular alterations that may induce overexpression of these substances' receptors in the dorsal root ganglion, generating pain (82). The exact mechanisms of myalgia, despite a few hypotheses that have been published, are still not completely understood. Thus, in view of the considerable presence of myalgia as a clinical manifestation of COVID-19, further investigation of the musculoskeletal condition and its association with SARS-CoV-2 is yet needed.

#### Myositis and Subtypes

There have been a few case reports indicating myositis as a manifestation of COVID-19. To date, ~23 cases related to SARS-CoV-2 infection have been described (83). The prevalence of the condition varies between 11 and 50% among scientific studies (84). It has been determined clinically that acute viral myositis has the potential of presenting as a sole manifestation of COVID-19 infection without respiratory symptoms (85), which indicates the importance of studying the disease.

Myositis secondary to COVID-19 may manifest in multiple forms, which range from muscle weakness to typical dermatomyositis identified mostly by classic rashes, or sheer back pain (83).

The first MRI-proven case of myositis induced by COVID-19 was reported in April 2020 in France (84). In this patient, autoimmune myositis was hypothesized due to the demonstrated association of myositis followed by interstitial pneumonitis in a COVID-19 patient, even though further immunological tests searching for any forms of myositis were negative. This has led to the conclusion that the association between muscle inflammation and interstitial pneumonia can be found in either COVID-19 or autoimmune myositis (84). Dermatomyositis, for instance, has been indicated as a potential trigger of interstitial lung disease, especially in anti-MDA5 (anti-melanoma differentiation-associated gene 5) antibody-positive patients, which are at higher risk for developing interstitial lung disease (83). This makes it difficult to differentiate dermatomyositis from interstitial lung disease and associate it with COVID-19 acute respiratory distress syndrome in SARS-CoV-2 infected patients (86).

On the other hand, it has also been suggested that viral infections can spur the development of autoimmune disorders such as myositis through myositis-specific antibodies. Along with this finding, a steep increase in dermatomyositis cases between April and August 2020, a period concurrent with the COVID-19 pandemic, has been reported in the city of Mumbai, India (86). With a few other studies outlining the inter-relation between dermatomyositis and COVID-19, it has been stated that dermatomyositis might be another musculoskeletal manifestation of SARS-CoV-2 infection (83).

Dermatomyositis is commonly presented as typical raches, such as malar erythema, heliotrope with periorbital edema, or diffuse facial rashes associated with symmetric and proximal muscle weakness. Diagnosis of dermatomyositis normally includes the identification of myositis-specific autoantibodies, such as anti-Mu2, anti-MDA5, anti-SAE1 (anti-small ubiquitin-like modifier-1 activating enzyme), or anti-nuclear autoantibodies (87).

A series of case reports in Mumbai, India indicated that MDA-5 may drive the production of great amounts of type I interferons, which contributes to the innate immune response against viruses (86). As a viral sensor, MDA-5 is activated by viral RNA.

It is known that immune response against coronavirus usually encompasses IFN induced with helicase C domain protein 1 (IFIH1) production, whose gene is a target of anti-MDA5 antibodies, which would contribute to an increase in MDA-5 production during viral infection, intensifying immune responses against viruses. Experts around the world have been examining COVID-19, for instance, as a possible human model of anti-MDA5 Idiopathic inflammatory myopathy (IIM), which might enhance COVID-19 treatment development: high-dose corticosteroids, human immunoglobulins, Janus kinase (JAK) inhibitors, and T cell modulators currently in trials and IL-6 inhibitors (e.g., tocilizumab), anti-GM-CSF (gimsilumab), IL-1 inhibitors (e.g., anakinra), and anti-IFNγ agents (e.g., emapalumab) could, thus, be considered as a potential treatment of COVID-19 (88).

As indicated by the series of case reports in Mumbai, India, MDA-5 may drive the production of great amounts of type I interferons, which not only contributes to the innate immune response against viruses (86) but also accounts for autoimmune triggers in dermatomyositis (89). Moreover, an increase in type I interferons is also seen in coronavirus infections, due to the fact that IFIH1 augments the production of cytokines such as IFN-γ, IL-1β, TNF-α, IL-6, and IL-18 (86), which would contribute to autoimmune disease emergence. This was recently demonstrated in dermatomyositis patients in 2020 which identified six distinct epitopes with high sequence identity to the human SARS-CoV-2 virus. Three of the linear epitopes of six amino acid lengths were indicated as highly specific for SARS-CoV-2 (87). Thus, it is possible to assume that SARS-CoV-2 infection may contribute to musculoskeletal autoimmune disease development.

Furthermore, a significant expression of MHC class I antigens on the sarcolemma of COVID-19-patients in the early phase of the disease has been detected. Additionally, an upregulation of MHC class II on myofibers antigens in later stages of the viral infection has also been identified in these patients, which reinforces skeletal muscle's involvement in the immune response against SARS-CoV-2 (90).

#### Rhabdomyolysis

Rhabdomyolysis has been indicated by the scientific literature as a possible manifestation of COVID-19. The condition is a syndrome that stems from skeletal muscle damage, which triggers the release of intracellular muscle proteins and enzymes into the bloodstream (91), or from a failure of energy production, which increases intracellular calcium, causing cellular lysis (92). Symptoms of rhabdomyolysis encompass myalgia, muscle weakness, fatigue, and dark-colored urine. Elevated concentrations of creatine kinase, lactate dehydrogenase, transaminases, and myoglobin levels are commonly detected in the syndrome (91, 93). Rhabdomyolysis can be caused by trauma, exertion, medications and drugs, myopathies and metabolic syndromes, and infections, including infectious myositis (92).

During the COVID-19 pandemic, diagnosis of rhabdomyolysis has been hampered mainly because both rhabdomyolysis and COVID-19 are characterized by fatigue, myalgia, and elevated levels of liver enzymes and lactate dehydrogenase. To date, only two cases of rhabdomyolysis secondary to COVID-19 have been reported. Thus, it is of critical importance to investigate the inter-relation between rhabdomyolysis and SARS-CoV-2 infection so as to differentiate the conditions and treat them both adequately. A recent case report of COVID-19 induced rhabdomyolysis has demonstrated that creatine kinase levels > 5 times the normal value in patients hospitalized with COVID-19 might be indicative of rhabdomyolysis, which would contribute to medical professionals failing to identify and diagnose rhabdomyolysis when COVID-19 is present (91).

Mechanisms of rhabdomyolysis induced by acute viral infection remain unclear. They may be associated with **myotoxic cytokines** that have the potential to cause immune-mediated injury and direct viral invasion damage. It has been indicated that a general infection causing rhabdomyolysis might be related to **direct injury** established by the pathogen or an **exacerbated inflammatory response** (93). Myotoxic substances as a treatment for SARS-CoV-2 infection may also induce side effects that are correlated to rhabdomyolysis (93). Immune cross-reactivity between antigens and myocytes, deposition of antigen-antibody complexes, and viral transformation of host cells and host proteins might also prompt an exaggerated immune response and muscle damage associated with rhabdomyolysis (92).

Rhabdomyolysis triggered by SARS-CoV-2 infection is of critical significance. The debilitating effects of the condition on patients, especially those that are already experiencing COVID-19 and its associated morbidity, pose a serious threat to these individuals' lives. Therefore, in order to enhance patient outlook, further studies on the inter-relation between the two conditions are absolutely required.

#### Skeletal Muscle Atrophy

Many patients that experience COVID-19 are required to undergo invasive mechanical ventilation to avoid a complete collapse in gas exchange, respiratory muscle fatigue, organ failure, and, ultimately, death. Mechanical ventilation associated with critical SARS-CoV-2 infection plays a relevant role in the deterioration of respiratory muscle structure and function, especially the diaphragm muscle, the main inspiratory muscle (11).

Mechanical ventilation induces a process of partial or complete unloading of respiratory muscles' activity, which leads to weakness, and silences the respiratory control centers in the brain stem (11). Regarding the diaphragm muscle, its inactivity during mechanical breathing is the main contributor to weakness (94), characterized by reduced motion, thinning, and decreased capacity to generate pressures in response to the phrenic nerves' stimulus. Inactivity of this muscle has also been associated with the abundant generation of ROS, apoptosis activation via caspase-3 expression, and upregulation of mRNA coding for ligands that are associated with the proteolytic ubiquitin-proteasome pathway (95). Inflammatory mediators play a causative role in diaphragm dysfunction, due to the activation of proteolytic pathways and an increased amount of neutrophils and macrophages (11).

These findings are of critical importance to severely ill SARS-CoV-2 infected patients since constantly evaluating mechanical ventilation time could potentially contribute to reducing COVID-19's detrimental effects on patients. Decreasing the time to which patients are exposed to mechanical ventilation may limit the destructive potential of the process on respiratory muscles and functional status (11).

### Musculoskeletal Neuromuscular Complications Due to COVID-19

#### Peripheral Neuropathy

Peripheral neuropathy refers to a group of disorders that affect the peripheral nervous system (96). Patients normally present with sensory abnormalities and autonomic dysfunction. The condition is usually associated with increased morbidity, especially due to the presence of neuropathic pain, weakness, falls, and disability in individuals affected by it. Patients usually undergo sensory abnormalities, and autonomic dysfunction (97).

Peripheral neuropathy and COVID-19 seem to be correlated, due to the fact that there has been a progressive amount of case reports of peripheral neuropathy in SARS-CoV-2 infected patients (98, 99). The condition has been reported to stem from a few conditions, mainly compressive neuropathies, symmetric polyneuropathy, mixed central and peripheral nervous system disorders, and systemic effects from critical illness neuropathy (100).

The influence of SARS-CoV-2 on the nervous system depends upon virus entrance into the system. Although the exact mechanism of entry into the central nervous system (CNS) is still undetermined, studies have suggested that virus routes may include retrograde neuronal transport across infected neurons, entry via the olfactory nerve's neuroepithelium (101), entry via the sensory fibers of the glossopharyngeal, entry *via* peripheral nerve (102), infection of the white blood cell or vascular endothelium travel across the blood-brain barrier (100, 103). It was also hypothesized that the virus might have direct access to the CNS, due to the fact that there have been two cases reporting SARS-CoV-2 in the cerebrospinal fluid (CSF) (104). This would be explained by the presence of ACE2 on neurons and glial cells in the CNS (105). Additionally, it has been indicated that SARS-CoV-2's typical cytokine storm may play a role in neuropathy development (99). Additionally, side effects of drugs used to treat COVID-19 symptoms and compression of peripheral nerves due to prolonged ICU-bedding in patients infected by the virus might also contribute to peripheral neuropathy in the setting of COVID-19 (12).

Peripheral neuropathy in patients infected by SARS-CoV-2 has been a challenge both to the healthcare professionals and to the patients themselves. Uncertainty regarding the relationship between the diseases has been hampering the ability of these professionals to generate better medical end results for the patients. Thus, it is of extreme importance to investigate the conditions in a more thorough manner, especially through clinical studies, which would contribute not only to improved clinical outcomes but also to enhanced treatment.

#### Guillain-Barré Syndrome and Subtypes

Guillain-Barré Syndrome (GBS) is an autoimmune neurologic disease comprised of a spectrum of polyneuropathies (102) characterized by ascending muscle weakness associated with decreased or absent deep tendon reflexes, mild to moderate sensory loss, occasional cranial nerve involvement, and radicular or muscle pain. The syndrome is usually demyelinating, but it can also involve primary axonal injury [sensory axonal neuropathy (AMSAN) and acute motor or acute motor axonal neuropathy (AMAN)] (106). The most common subtype of the disease is the acute inflammatory demyelinating polyneuropathy (AIDP), AMAN, and the Miller Fisher syndrome (MFS) characterized by acute ophthalmoplegia, gait ataxia, and areflexia (102).

The condition is normally triggered by viral infections, which is supported by the fact that in 70% of cases, neurologic symptoms' advent may depend on the precedent occurrence of viral illness (106). General coronaviruses, for instance, already had their involvement with neurological disease demonstrated by scientific literature. There have been recent reports of GBS possibly triggered and even caused by COVID-19 (101). Numerous cases of the neurological syndrome post-COVID-19 have been reported to follow either a para-infectious or a post-infectious pattern (107), para-infectious being more common (108).

SARS-CoV-2 molecular mechanisms involved in COVID-19 nerve damage are still not clear, even though there are a few hypotheses that have been proposed, as previously stated herein. Particularly in GBS, SARS-CoV-2 infection could drive the production of antibodies against specific antigens associated with the neurological disease (109) (Figure 1). It is also likely that the cytokine storm triggered by COVID-19 takes part in the development of GBS in SARS-CoV-2 infected patients, as it has been reported that cerebrospinal fluid of patients with GBS is characterized by elevated IFN-γ and their serum, characterized by elevated TNF-α, which are cytokines that are also peaked in the setting of COVID-19 (108). Previous systematic reviews of GBSs cases in the context of COVID-19 have demonstrated that SARS-CoV-2 infection symptoms usually precede GBS manifestations at a median interval of 14 days. This 2-week interval between both conditions coincides with the second phase of COVID-19, marked by the cytokine storm, which would reinforce the participation of COVID-19 cytokines in the pathogenesis of GBS in SARS-CoV-2 infected patients. In this phase, critically ill patients suffering from COVID-19 may manifest GBS in a masquerade form as a mere critical illness polyneuropathy. Thus, difficulty in weaning from mechanical ventilation may be a consequence of GBS (110).

Apart from that, it has been suggested that SARS-CoV-2 entrance on cells depends upon S protein binding not only to ACE2 receptor but also to glycoproteins containing sialic-acid and cell surface gangliosides, which would interestingly increase viral transmissibility (111). Gangliosides are sialylated glycosphingolipids located profusely in several nervous tissues. Antiganglioside antibodies commonly develop post-infection (typical GBS course in the classical form of the disease) (108) since gangliosides and infectious agents share epitopes, which leads to a molecular mimicry phenomenon: binding of the antibody to nerves, which causes impaired conduction of impulses and axonal degeneration (108). That is, the antibodies formed against the viral glycoproteins act upon glycoconjugates on the neural tissue, mainly myelin, and axon of peripheral nerves, impairing it (75).

Copious gangliosides, mainly those that contain a disialosyl moiety (GQ1b, GT1b, and GD1b, for example) or 2 gangliosides that share epitopes with GM2, or a combination of GM1 and GM2, GD1b and GM1, can work as antigens in patients with neuropathies (112). When IgM recognizes the Gal (pl-3) GalNAc moiety of GM1, which is found on the surface of motor neurons, a motor neuropathy typically occurs, and when it recognizes epitopes that contain disialosyl groups of GDlb (located on the dorsal root of ganglionic neurons), patients present with sensory ataxic neuropathy (112). SARS-CoV-2 S protein probably interacts with the GalNAc residue of GM1 and dimers of ganglioside for anchoring to cell surface gangliosides. This process is likely to generate cross-reactivity between epitopes within SARS-CoV-2 gangliosides that bear S protein and surface peripheral nerve glycolipids (102), which act as a mechanism in SARS-CoV-2 triggered autoimmune GBS. In accordance with that is the report of numerous GBS variants in SARS-CoV-2 in the scientific literature (106).

As a rare but potentially fatal neurological condition, primarily due to swiftly progressive weakness of limb, bulbar, and respiratory muscles (108), special attention should be given to GBS development in the setting of COVID-19. Early diagnosis and management can improve clinical outcomes. Considering the yet unclear mechanisms of GBS emergence and SARS-CoV-2 infection, more extensive studies are still needed (109).

## Mitigation of SARS-CoV-2 Impact on Skeletal Muscle

The detrimental effects of COVID-19 on skeletal muscle, herein previously outlined, are characterized by a variety of causes, especially inflammation. Because COVID-19 recovery poses a multidimensional challenge, different aspects of the disease should be analyzed: for instance, from pre-infection up to moderate illness, strategies that address viral replication and immune system fortification should be examined, while in more severe cases, anti-inflammatory strategies need to be the focus (113). In order to mitigate the burden caused by SARS-CoV-2 infection on skeletal muscle, a few of those strategies should be mentioned.

**Figure 3:**
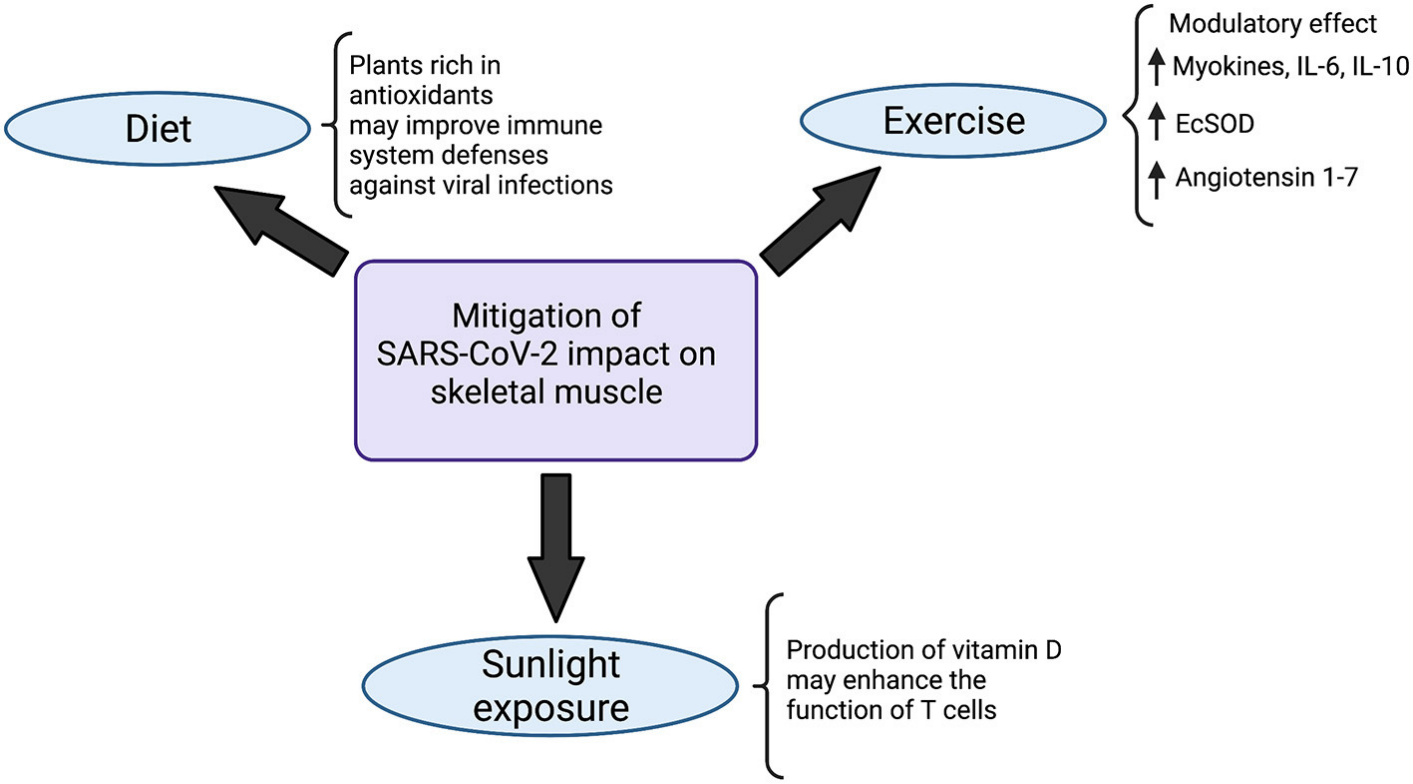
Strategies to mitigate SARS-CoV-2 impact on the musculoskeletal system. Diet modifications, sunlight exposure and exercise have been indicated in the literature as potential strategies to minimize the impacts of SARS-CoV-2 on skeletal muscle. Meals rich in antioxidant plants likely boost defenses of the immune system, improving viral infections combat. At the same time, the production of vitamin D, triggered by exposure to sunlight, enhances the function of T cells. Physical activity, on the other hand, exerts a modulatory effect in the organism, mainly characterized by a state of increase in myokines, IL-6 and IL-10, which improves the body's immunovigilance. Augmented expression of antioxidant Extracellular Superoxide Dismutase enzyme (EcSOD) has also been detected during exercise, which contributes to both oxidative stress and tissue damage reduction. Angiotensin 1-7 also seems to be upregulated in the setting of physical activity, which plays a role in mitigating the deteriorating effects of SARS-CoV-2 in the organism. Created with Biorender.com.

Initially, studies have indicated some diet alterations that might act in favor of the immune system. Meals filled with plants rich in antioxidants, for instance, may improve the immune system defenses against viral infections (114, 115) (Figure 3): blueberry, for example, has been shown to increase the amount of NK cells and anti-inflammatory cytokines, upon acute ingestion (116). In contrast with that, processed oils (as opposed to short-chain fatty acids), for instance, shall be avoided, due to the demonstrated enhancement of a more proinflammatory state when ingested (117). Avoiding alcohol should also be recommended since a few studies have demonstrated that it jeopardizes not only the function of key immune cells, such as macrophages and neutrophils in the alveoli, but also the barrier structure of lower airway epithelia.

Besides diet modifications, moderate sunlight exposure also seems to play a role in immunity enhancement: the production of vitamin D in the skin, stimulated by sunlight, may enhance the function of T cells (118–120) (Figure 3).

Exercise is another practice that acts in favor of the immune system, playing a pivotal role in boosting it (121). Moderate physical activity is associated with a higher intensity of macrophage anti-pathogenic activity and elevations in anti-inflammatory cytokines, which would contribute to alleviating the effects of pathogens on the organism (122). During exercise, inflammatory response and stress hormones are normally decreased whereas lymphocytes, NK cells, immature B cells, monocytes (123) and naive T cells (123) are increased, which, on the whole, determines an improvement in immunovigilance (123). The modulatory effect of physical exercise has also been demonstrated in a few studies by an increase in myokines, such as myostatin, IL-6, IL-10 and leukemia inhibiting factor (124) (Figure 3). Importantly, even though IL-6 participates intensely in acute inflammation, the cytokine has also been shown to exert anti-inflammatory effects (125). Studies regarding aerobic exercise detected diminished levels of senescent/exhausted CD4 and CD8 T cells (126) and reduced immunosenescence (127), which is positively associated with the quality and quantity of antibodies against SARS-CoV-2 (125, 128). Besides that, during physical activity, enhanced expression of the antioxidant Extracellular Superoxide Dismutase enzyme (EcSOD) has also been detected. EcSOD plays an important role in oxidative stress and tissue damage reduction in the setting of COVID-19 (129) (Figure 3).

Additionally, an exchange of white blood cells between the tissues and the circulatory system takes place during regular exercise, which is responsible for reducing morbidity and mortality associated with acute respiratory illnesses and viral infections (130). Exercise, particularly the ones that overload the cardiorespiratory system, also generate a state of mobilization and redistribution of effector lymphocytes, which is mediated by catecholamines. These lymphocytes then migrate to structures such as the upper respiratory tract, lungs and intestines, where they recognize and combat pathogens, improving response against viruses (131). Studies that analyzed Influenza infection indicated that moderate exercise is an important factor in viral load and inflammatory response reduction (132) (Figure 3).

On top of that, exercise seems to prompt an upregulation of lung ACE receptor, which accounts for an increase in the production of angiotensin 1-7. Considering the protective effects of angiotensin 1-7 in the organism, it could be stated that exercise contributes to a mitigation of the harmful effects of SARS-CoV-2 in the lungs (133). Considering that the respiratory tract is the source of entrance of the virus, reducing the effects of viral infection in the lungs could also exert a protective role on the rest of the organism (Figure 3).

Overall, good physical conditioning has been correlated with a reduction in risk of latent viral infections reactivation, characterizing a better immune system dynamic (131). Aerobic activity has been associated with a significant decrease in severity and incidence of symptoms of upper respiratory tract infections (URTI) (134).

Importantly, intense physical activity before or during an infection such as COVID-19 and influenza is not recommended, since it can precipitate severe disease (123, 135) due to the immunosuppression induced by Th2 anti-inflammatory cytokines aimed at reducing muscle tissue damage (122, 135). Therefore, intense exercise should be avoided until normalization of the symptoms and end of the disease (136). In conclusion, it is possible to state that as a potential enhancer of the immune system, physical exercise could be pointed as an important factor for protection against the deleterious effects of SARS-CoV-2 on the human body.

## Bioengineering and Skeletal Muscle Models

Given the current uncertainty that exists around the long-term impact of SARS-CoV-2 infection on skeletal muscle, there is a need to establish a body of research that directly interrogates muscle tissue dynamics during both SARS-CoV-2 infection and recovery. Traditionally, skeletal muscle biology is studied in 2D cell culture, animal models, or through direct sourcing *via* biopsies from healthy and diseased individuals. Though samples isolated from human subjects are ideal, ethical considerations and low availability of tissue limit the scope of studies able to be performed. Despite the fact that animal models have been frequently utilized, both replicability and translatability to human disease are relatively poor (137–139). 2D cell culture of immortalized muscle cell lines induced to myotubule differentiation through serum starvation is also commonly applied in rapid drug screening assays (140). However, despite the benefits of being able to control differentiation and precisely monitor cell microenvironment, the myotubules formed are developmentally immature with limited physiological relevance and thus the information gleaned is likewise limited in kind.

Physical features of stiff 2D culture plates have been found largely at fault for this functional immaturity, shortening culture duration and decreasing muscle-specific gene expression. In contrast, substrates with similar stiffness of 12–18 kPa to that of native skeletal muscle tissue have demonstrated improved myotubule structure (137, 141). As a result, great strides have been made to create biomimetic 3D muscle culture to better study muscle dynamics in drug and pathology research *in vitro*. Compared to 2D muscle cell culture, 3D culture allows for increased myotube size, longer culture times, and protein content, and improved maturation of the key skeletal muscle gene myosin heavy chain (MHC) (142–144).

The field of bioengineering is continually playing a promising larger role in the study of muscle pathology and regenerative medicine on the preclinical level as 3D biomimetic cultures are being further refined. 3D cultures with the goal of studying muscle dynamics should use proper cells and signals within a matrix that (1) accurately replicates structure function and regenerative capacity of native structures, (2) represents the phenotype of the disease of interest, and (3) is able to be monitored by the most current functional and metabolic technologies (137, 145).

### Biomaterials for 3D Skeletal Muscle Modeling

Biomaterials are material systems that have been engineered to direct the course of a diagnostic or therapeutic procedure by controlling interactions with components of living systems either alone or in part of a system (146). The term biomaterial is a broad classification of either naturally sourced or synthetically generated materials that have applications ranging from *in vitro* modeling to clinical tissue engineering systems for regenerative medicine.

Functional *in vitro* skeletal muscle models as summarized in Table 1 provide a biomimetic microenvironment with tissue-specific ECM qualities and mechanical and biochemical signals that promote accurate tissue function and rapid tissue maturation. Biomaterials utilized must be biocompatible, have high surface area for cell interaction and adhesion activity, provide mechanical integrity over both short and long terms, and must be diffusion capable to meet cellular nutrient and gas transport needs.

**Table 1:** Bioengineering skeletal muscle models.

**Model**	**Benefits**	**Limitations**
Synthetic ECM	*Chemically sourced* • High replicability • Standardized physical properties of source material • Ability to be formed into unique geometries • Precise tuning of final structure mechanical properties • Enhanced myoblast alignment and differentiation • Ease of chemical modification	*Chemically sourced* • Potentially caustic degradation products • Biocompatibility issues • Immunogenicity
	*Naturally sourced* • Generally biocompatible • Safe degradation products • Naturally have (or have the ability to) link growth factors • Enhanced myotubular alignment • Muscle cell growth stimulation	*Naturally sourced* • High prevalence of batch variability • Mixed endogenous remodeling capabilities • Native ECM secretion stimulation is highly dependent on the material selected • Low contractile forces • Difficulty stimulating muscle cell maturation
Decellularized tissue	• Native tissue architecture is maintained (basement membrane architecture, vascular networks) • Endogenous bioactive molecules are present (growth factors, glycosaminoglycans) • Mechanical properties of source tissue are maintained • Supports innervation	• Size limitations • Lack of homogeny in decellularization • Volumetric limitations
Scaffold free assembly and organoids	• Capable of long-term cell culture • Multi-lineage cell culture supportive • Quantifiable means of determining muscle activity and dynamics • Able to recapitulate some specific physiological structures, albeit on a smaller scale	• Size limitations • Long-term culture requirements • Biological transport issues • Scale-up feasibility
Microfluidics and microchips	• Precise control of culture microenvironment • High throughput • Utility in drug screening • Capable of long-term cell culture • Can stimulate skeletal muscle differentiation and maturation • Quantifiable means of determining muscle activity and dynamics • Multi-lineage cell culture supportive	• Lack of 3D tissue architecture • Limited geometries • Highly complex assembly • Scale-up feasibility

*The benefits and limitations associated with bioengineering skeletal muscle modeling systems*.

#### Synthetic ECM

The most up-front approach utilized in efforts to engineer and model skeletal muscle has been to synthetically generate ECM structures to better replicate muscle tissue microenvironments. A wide variety of biomaterials have been utilized as the basis for these structures, including both synthetically engineered and naturally derived material systems.

Synthetic materials have been frequently utilized for skeletal muscle regeneration, including polyethylene glycol (PEG), poly lactic-co-glycolic acid (PLGA), poly-l-lactic acid (PLLA), polyglycolic acid (PGA), their copolymers (PLLA/PLGA), and polycaprolactone (PCL) (147). Synthetic material systems have several advantages in their application, including overall replicability, physical property standardization, and the ability to be readily formulated into many different geometries and patterns. As a result, structural properties can be tuned with high precision to address case-specific needs (147). These characteristics have been able to enhance myoblast alignment and differentiation capabilities (148). Though synthetic materials have the potential of precise physical property tuning and are capable of cell supportive modifications, robust limitations to clinical application still exist—including their degradation products, suboptimal biocompatibility, and immunogenicity potential.

Biologically sourced material systems from plants and animals tend to display favorable biocompatibility and degradation byproduct profiles than their synthetic counterparts. Chitosan, silk fibroin, alginate, and agarose are all biologically derived and commonly applied in biotechnology industries (149–151). All of these compounds have been broadly applied in tissue engineering strategies because of endogenous bioactive properties that allow them to naturally link growth factors (147). For example, alginate hydrogels are able to assist in myoblast proliferation and maturation while also promoting the release of growth factors essential for muscle regeneration through polymer modification (148, 152–155).

Mammalian sourced material systems, such as collagen and fibrin, have the benefit of being naturally decorated with proteins and signaling molecules that muscle cells directly interact with. Collagen, type I collagen specifically, is the most abundant ECM protein in native skeletal muscle (137) and was also the first material system utilized to generate a 3D model of skeletal muscle in 1988—in which avian sourced myotubules were embedded into a type I collagen gel scaffold (143). 3D collagen scaffolds have also been shown to be supportive in rodent and human primary skeletal muscle myoblast culture applications and in the formation of engineered skeletal muscle tissue (156–158). Collagen scaffolds have demonstrated the ability to increase myotubular alignment and effectively stimulate growth (159–161). Though it has been shown to be useful, type I collagen also has limitations in skeletal muscle engineering endeavors. Collagen is not easily remodeled, does not stimulate ECM secretion, in high concentrations has resulted in low contractile forces and has adverse effects on muscle cell maturation (162, 163). Thus, though collagen was very frequently utilized in early skeletal muscle tissue engineering endeavors, other material systems have since been identified that usurp these critical shortcomings.

Fibrin is core to the healing process in animals as the major structural component in the blood-clotting pathway. Once a blood clot forms, it is structurally replaced with secreted cellular ECM during the healing process (164). It is due to this endogenous remodeling capability that fibrin is commonly applied in skeletal muscle modeling and tissue engineering endeavors, as muscle cells are able to degrade and replace the base fibrin scaffold with naturally secreted ECM (165). Additionally, fibrin has a stiffness similar to that of native skeletal muscle structures, unlike type I collagen, which is far stiffer (165–167). Beyond physical property similarities, fibrin also has demonstrated the ability to promote angiogenic and neuronal activity—therefore fibrin has great promise in generating more realistic tissue microsystems to better recapitulate the *in vivo* environment (168, 169). Like any other naturally sourced system, batch-to-batch variability is still a challenge, however, this can be mitigated through testing and controls (144).

#### Decellularized Tissues

Compared to scaffolds consisting of one or two biopolymers engineered to mimic native ECM structures, there is also the option to apply native ECM structures directly. Compared to engineered scaffolds, decellularized scaffolds maintain native microstructures, bioactive molecules, and vascular networks while similarly maintaining the mechanical properties of the tissues from which they are sourced (170, 171). As a result of having native skeletal muscle growth factors, basement membrane structural proteins, and glycosaminoglycans, myogenic cell proliferation is supportive and *in vivo* remodeling in response to seeded decellularized scaffold application is feasible (171). Biologic scaffolds have also demonstrated the ability to support skeletal muscle reconstruction through significant vascularization and innervation and the formation of both type I and type II muscle fibers (172). Even greater regenerative capacity is seen with the uniaxial stretching (173) and the application of these decellularized tissues with stem or progenitor cells (174). However, there exist several limitations in the execution of this technology—including the lack of homogeny in decellularization in larger, thicker tissue samples such as skeletal muscle and critical volumetric limitations in their application in general (174). Despite these limitations, greater strides in perfusion decellularization techniques and co-application with cell therapies have made volumetric muscle loss repair more translationally feasible (175).

#### Scaffold-Free Assembly and Organoids

Self-assembled 3D tissues can also be formed through endogenous ECM secretions without the utilization of material systems. Self-assembled skeletal muscle tissue has been assembled through a variety of means, including the utilization of non-cell supportive membranes (176), micropatterning (177–180), and monolayer layering (181–183) in either pure or co-culture with fibroblasts or other viable cell lineages. Organoids have the potential of generating microscale systems for monitoring skeletal muscle activity over longer terms that can span months (184). In addition to skeletal muscle cells, skeletal muscle organoids can contain cells of different lineages for monitoring specific physiological structures, such as neuromuscular junctions (184). Recent advancements in the creation of vascularized skeletal muscle organoids have been providing a possible solution to current organoid size limitations (185). In addition, fully human, multilineage skeletal muscle organoid models that contain all lineages essential to skeletal muscle, including endothelial cells, pericytes, and motor neurons have also been recently successfully generated with the use of iPSCs (186). Skeletal muscle organoids have also been shown to be capable of being utilized in force generation studies as indicators of muscle activity (187). Limitations of scaffold-free assemblies and organoids are long-term culture requirements, small tissue size, biological transport concerns, and challenges with the scale-up feasibility of the technology (137).

#### Microfluidics and Microchips

Cell culture through the application of microchip and microfluidic systems, colloquially called “organ on a chip” devices, provides the ability to precisely control culture microenvironment for higher throughput analysis of cellular response and behavior and has great promise in drug screening and disease models (188). Regarding culture duration, 3D microchip cultures of skeletal muscle cells have been used as a viable tool for longer-term skeletal muscle cell culture (189). Microchip models have been able to recapitulate many aspects of skeletal muscle cell activity—including contractibility upon external electrical stimuli (190), skeletal muscle cell migration activity (191), and differentiation into multinucleated skeletal muscle tissue bundles (189, 192). Micropatterning microgrooves into microchip culture demonstrated the ability to initiate cytoskeletal and nuclear alignment in a directionally specific manner for more relevant physiological models of skeletal muscle (193). Microfluidic chips have also been able to allow motor neurons to form neuromuscular junction synapses with skeletal muscle cells (194). Limitations of microfluidic and microchip technologies are that there is a lack of native 3D tissue architecture, limited and complex system geometries, and scale-up fabrication issues (195).

## Conclusion and Future Directions

COVID-19 is still a reality that severely impacts society, particularly patients and healthcare professionals. Uncertainty regarding the exact mechanisms that explain the wide range of symptoms and secondary diseases experienced by individuals infected by SARS-CoV-2 has been preventing the medical field from providing adequate treatment to patients. Of special importance in this matter, as shown in this review, is the musculoskeletal system, due to the progressive reports of aggressive skeletal muscle complications associated with COVID-19, mainly sarcopenia, cachexia, rhabdomyolysis, peripheral neuropathy, and Guillain-Barré syndrome. It is believed that these conditions are prompted predominantly by the cytokine storm triggered by SARS-CoV-2, but a few studies have also indicated an association with ACE2 downregulation, hypoxia, direct viral invasion into the skeletal muscle, and physical inactivity. Further research to determine the correlation between these conditions and SARS-CoV-2 infection could drive the development of tailored treatment plans that could mitigate or prevent severe musculoskeletal deterioration in those affected by the virus. Importantly, *in vitro* 3D cell culture models are appealing strategies to study the long-term impact of SARS-CoV-2 infection on skeletal muscle. They provide a biomimetic microenvironment with tissue-specific ECM qualities. Furthermore, the interaction among cells and cells with ECM is similar to the natural environment found *in vivo*. Several bioengineering models were reviewed in this work such as synthetic ECM, decellularized tissues, scaffold-free assembly, organoids, microfluidics, and microchips. Future research should be associated with these technologies to understand the skeletal muscle complications after SARS-CoV-2 infection.

## Author Contributions

RS, RU, CP, and ES: conceptualization and funding acquisition. RS: supervision. MS, LM, SS, GL, RS, and CB: writing—original draft. MS, LM, SS, GL, RS, CB, RU, CP, and ES: writing—review and editing. CB and SS: design and prepare figures. All authors contributed to the article and approved the submitted version.

## Funding

The authors thank the Fundação de Amparo à Pesquisa do Estado de São Paulo - FAPESP: 2016/20796-2 (RU), 2020/04709-8 (RU), 2018/06088-0 (CP), and 2019/10922-9 (RS); Coordenação de Aperfeiçoamento de Pessoal de Nível Superior - CAPES: code 001 (CB), Conselho Nacional de Desenvolvimento Científico e Tecnológico - CNPq: 303035/2018-8 (CP), and 405691/2018-1 (CB); FAPFCMSCSP 2019/2021); and American Heart Association - grant #19IPLOI34760654/ES/2019 and grant #20PRE35210399/SS.

## Conflict of Interest

The authors declare that the research was conducted in the absence of any commercial or financial relationships that could be construed as a potential conflict of interest.

## Publisher's Note

All claims expressed in this article are solely those of the authors and do not necessarily represent those of their affiliated organizations, or those of the publisher, the editors and the reviewers. Any product that may be evaluated in this article, or claim that may be made by its manufacturer, is not guaranteed or endorsed by the publisher.
